# Intraoperative assessment of myocardial perfusion using near-infrared fluorescence and indocyanine green: A literature review

**DOI:** 10.1016/j.xjtc.2025.01.015

**Published:** 2025-01-28

**Authors:** Roderick C. Peul, Rohit K. Kharbanda, Stefan Koning, Mo W. Kruiswijk, Floris P. Tange, Pim van den Hoven, Alexander L. Vahrmeijer, Robert J.M. Klautz, Jaap F. Hamming, Jesper Hjortnaes, Joost R. van der Vorst

**Affiliations:** a Department of Surgery, Leiden University Medical Center, Leiden, The Netherlands; b Department of Cardiothoracic Surgery, Leiden University Medical Center, Leiden, The Netherlands; c Department of Cardiothoracic Surgery, Amsterdam University Medical Center, Amsterdam-Zuidoost, The Netherlands

**Keywords:** near-infrared fluorescence imaging, indocyanine green, myocardial perfusion, coronary artery bypass grafting, graft patency, quantification

## Abstract

**Background:**

Coronary artery bypass grafting (CABG) is among the most commonly performed major surgical procedures worldwide. While flow measurements help assess graft patency during surgery, there are limited tools available for surgeons to objectively evaluate myocardial perfusion after graft placement. Near-infrared fluorescence (NIRF) imaging shows promise in this area, offering real-time visualization of flow and perfusion without the need for radiation or nephrotoxic contrast agents. This review summarizes current knowledge of and developments in myocardial perfusion assessment via NIRF imaging, emphasizing the potential benefits of adding quantification to enhance this technique.

**Methods:**

PubMed was searched for articles describing the use of NIRF imaging for myocardial perfusion assessment. Articles were subsequently analyzed based on study objectives, subjects, and quantification capabilities. Limitations, future directions, and comparisons with other techniques were examined to recognize patterns and describe the chronological developments in NIRF imaging for myocardial perfusion assessment.

**Results:**

Twenty-eight articles were included, 11 of which explored quantification. Only 5 of these articles included patients. Aims and techniques varied significantly among studies. Compared to the abundance of qualitative assessments, quantified NIRF imaging in patients remains limited.

**Conclusions:**

This literature review highlights that NIRF imaging has been broadly researched qualitatively, showing promise for guiding CABG surgery through visualization of graft flow. However, the critical step of incorporating quantification to accurately assess myocardial perfusion remains insufficiently explored. To optimize decision making during CABG surgery, future studies must focus on intraoperative application of quantified NIRF imaging in cardiovascular patients.

**Figure:**
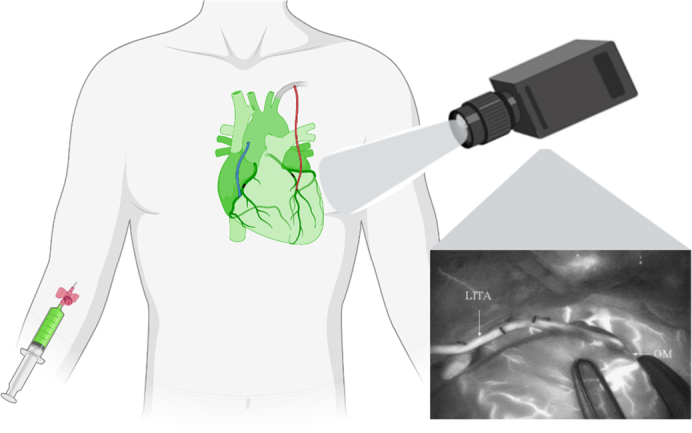
Simplified near-infrared fluorescence imaging during coronary artery bypass grafting.

**Central Message**

Current research shows that near-infrared fluorescence (NIRF) imaging aids qualitative graft assessment. Future studies should focus on intraoperative, quantified NIRF imaging for decision making regarding coronary artery bypass grafting.

**Perspective**

Cardiothoracic surgeons currently lack adequate tools to assess myocardial perfusion during coronary artery bypass grafting surgery, making it challenging to estimate clinical success intraoperatively. Near-infrared fluorescence is a feasible technique for assessing tissue perfusion, making the development of a quantified approach for real-time, objective evaluation of myocardial perfusion highly desirable.

Coronary artery bypass grafting (CABG) is one of the most commonly performed major surgeries worldwide, with several major developments through the years.^1^ This allows surgeons to perform bypass procedures both on-pump as well as off-pump.^2^^,^^3^ Contrast angiography remains the scarcely used golden standard for graft patency. Despite its accuracy, the technique is invasive and costly, and hospitals must be equipped with hybrid operating rooms. In addition, the use of harmful radiation and a possibly nephrotoxic contrast agent is necessary.

To avoid these problems, surgeons can make use of flow measurements to objectively measure flow through the graft.^4^^,^^5^ However, a major limitation of contrast angiography and flow measurements is that they provide an assessment of flow only in the graft itself, without providing information on perfusion of the myocardial tissue supplied by this graft. A patent graft does not necessarily translate into improved myocardial perfusion, as microvascular damage also can impair tissue perfusion. This poses a challenge in adequately predicting clinical improvement following graft placement. Therefore, surgeons are in need of a tool that not only evaluates graft patency, but also provides a more comprehensive evaluation of actual myocardial perfusion after CABG.

Near-infrared fluorescence (NIRF) imaging has demonstrated promising results in evaluating microperfusion across various tissues.^6^, ^7^, ^8^, ^9^, ^10^, ^11^, ^12^ The technique is based on the excitation of an intravenously administered fluorophore. The most commonly used fluorescent dye is the Food and Drug Administration–approved fluorophore indocyanine green (ICG). It is intravascularly bound to albumin and is feasible for superficial perfusion assessment because of low tissue autofluorescence in the specific spectral range (750-850 nm). With the use of a near-infrared camera, a fluorescent signal can be observed, visualizing blood flow. A higher fluorescence intensity corresponds to more blood in the area of interest. ICG is cleared rapidly by the liver and carries a low risk of side effects. Compared to other imaging modalities, NIRF imaging is a cost-effective technique.^13^ This is attributed primarily to the affordability of ICG and the relatively short duration of perfusion measurements, which typically range from seconds to several minutes, without the delay of patient transportation.

To date, however, most studies on myocardial NIRF imaging have provided only qualitative graft assessment, making interpretation subjective and potentially less accurate. In other tissues, recent techniques have demonstrated the possibility of quantifying fluorescent signal intensity, providing added value for the interpretation of perfusion for surgical applications.^14^ Combining the strength of quantification and the ability to assess both the graft and the perfused area with NIRF imaging might result in successful intraoperative, minimally invasive CABG assessment. In this literature review, we summarize the current knowledge and developments in myocardial perfusion assessment with NIRF imaging and highlight the potential added value of quantification to enhance this technique.

## Methods

### Search Strategy

PubMed was searched for studies reporting the use of NIRF imaging with ICG for myocardial perfusion assessment published up to June 2024. The search strategy was developed together with a certified librarian and combined the MeSH terms “Infrared Rays,” “Optical Imaging,” “Fluorescent Dyes,” and “Indocyanine Green” with “Myocardial Perfusion Imaging,” “Perfusion Imaging,” “Perfusion,” “Myocardium,” and “Thoracic Surgery”. The complete search strategy is provided in Appendix E1.

### Eligibility Criteria

Full-text English articles focusing on myocardial perfusion assessment by NIRF imaging were selected. Other assessment techniques or articles that merely mentioned the technique without an analysis of subjects were excluded. Articles that used identical data from previously published articles also were excluded, to minimize duplicate publication bias. Screening of the articles was conducted by 2 independent researchers (R.C.P. and R.K.K.). In cases of uncertainty, 2 other reviewers (J.H. and J.R.v.d.V.) were consulted.

### Data Collection

The selected articles were subsequently analyzed on the type of subjects used, aim of the study, and the possibility for quantification. Type of ICG administration and other measurement characteristics were compared as well. Limitations, future recommendations, and comparisons with other techniques were studied to identify patterns and create a chronologic timeline of the developments in NIRF imaging for myocardial perfusion assessment.

## Results

### Article Selection

**Figure 1:**
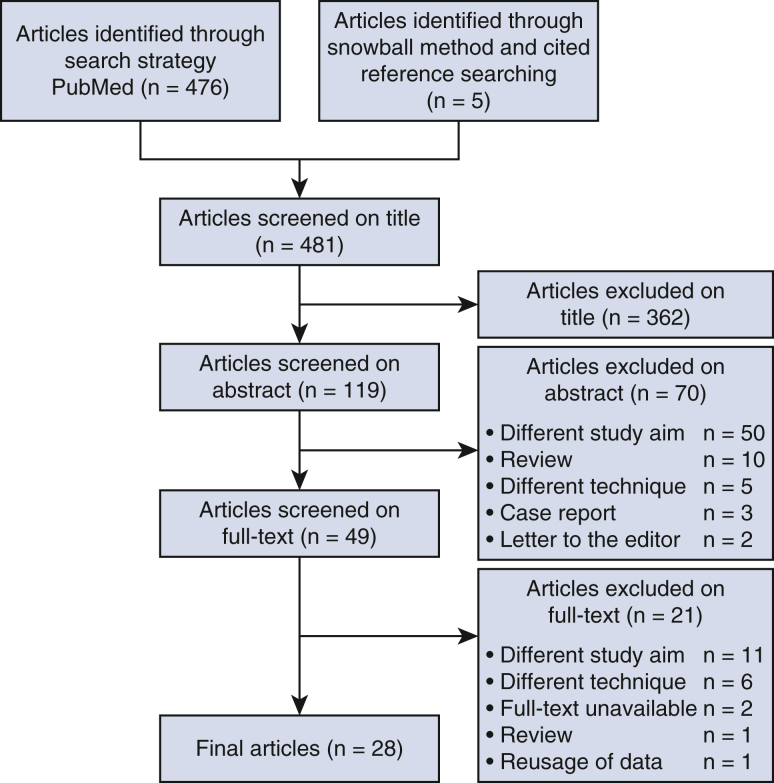
Article selection.

The search strategy yielded a total of 476 articles, of which 23 were deemed eligible for this review. A complementary search using the snowball method and cited reference searching resulted in an additional 5 articles, bringing the total number of articles to 28 (Figure 1).^15^, ^16^, ^17^, ^18^, ^19^, ^20^, ^21^, ^22^, ^23^, ^24^, ^25^, ^26^, ^27^, ^28^, ^29^, ^30^, ^31^, ^32^, ^33^, ^34^, ^35^, ^36^, ^37^, ^38^, ^39^, ^40^, ^41^, ^42^

### Subjects and Study Goal

Patients were the subjects in 20 studies; canine, murine, and porcine subjects were used in 1, 2, and 5 studies, respectively. The study goal varied among the articles, but the majority focused on graft patency in CABG surgery. Other articles focused on myocardial perfusion assessment, coronary anatomy, severity of coronary stenosis, delivery of cardioplegia, or a combination of these objectives.

### Qualitative Assessment

**Figure 2:**
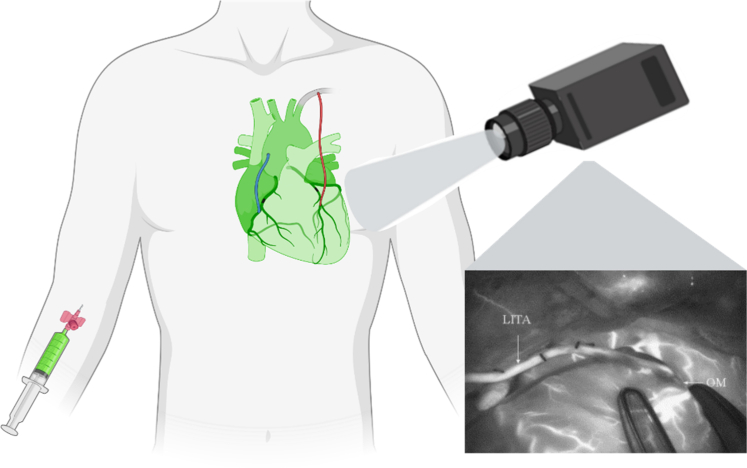
Simplified near-infrared fluorescence imaging during coronary artery bypass grafting. *LITA*, Left internal thoracic artery; *OM*, obtuse marginal artery.

**Figure 3:**
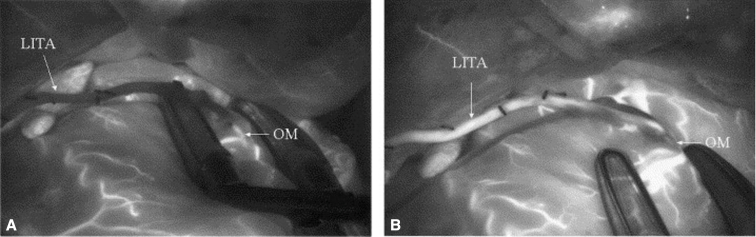
Qualitative assessment of graft patency with near-infrared fluorescence imaging.^19^ A, Absence of blood flow in LITA. B, Patent LITA after revision of anastomosis. *LITA*, Left internal thoracic artery; *OM*, obtuse marginal artery.

The first studies were conducted by Detter and colleagues^15^ and Rubens and colleagues^17^ in 2002 on graft assessment with NIRF imaging, involving porcine and human subjects undergoing CABG surgery. These studies focused on qualitative assessment and did not explore quantification possibilities. Subsequent years saw 14 studies on qualitative graft assessment with NIRF imaging in CABG patients^18^, ^19^, ^20^, ^21^, ^22^, ^23^, ^24^^,^^27^, ^28^, ^29^, ^30^^,^^33^^,^^35^^,^^39^ and 1 study in canine subjects by Hassan and colleagues.^32^ Most of the studies described the possibility of subjectively assessing graft patency with NIRF imaging and compared the technique to transit time flowmetry (TTFM) and coronary angiography. In 5 studies, the capability of visualizing the baseline anatomy of coronary arteries with NIRF imaging was described.^15^^,^^17^^,^^20^^,^^32^^,^^39^ Measurements were performed by adding ICG to either the cardioplegic solution in on-pump surgery or intravenously in off-pump surgery. Subsequently, the camera visualized flow through the graft, allowing surgeons to make a subjective estimate of graft performance and adjust grafts if deemed necessary (Figures 2 and 3). Compared to TTFM, with contrast angiography as the golden standard, the articles concluded that NIRF imaging was noninferior and in some articles even superior to TTFM for estimating graft performance.

Regardless of the promising results, 11 out of 17 articles mentioned the lack of quantification as a major study limitation, because of the subjective nature of the assessment. A complete overview of the articles describing qualitative assessment is provided in Table 1.

**Table 1:** Overview of articles describing qualitative NIRF for myocardial perfusion assessment

Author, y	Goal	Subject	Number	Study design	On/off- pump	Relevant findings
Detter et al,^15^ 2002	Coronary anatomy, coronary stenosis, graft patency	Pigs	23	Preclinical animal study	0/6	NIRF imaging is a feasible technique for visualizing blood flow in coronary arteries, bypass grafts, and myocardial tissue in animals.
Rubens et al,^17^ 2002	Coronary anatomy, graft patency	Patients	20	Clinical trial, phase II	18/2	NIRF imaging is a safe and feasible technique for visualizing coronary anatomy and bypass grafts in patients undergoing CABG.
Taggart et al,^18^ 2003	Graft patency	Patients	84	Clinical trial, phase II	19/65	NIRF imaging is a safe and feasible technique for visualizing coronary anatomy and bypass grafts in patients undergoing CABG.
Balacumarawami et al,^19^ 2004	Graft patency	Patients	200	Prospective observational study	45/155	NIRF imaging is capable of detecting failed grafts intraoperatively and allows for graft revision. No difference in the incidence of graft failure was seen between on-pump and off-pump surgery.
Reuthebuch et al,^20^ 2004	Coronary anatomy, graft patency	Patients	38	Prospective observational study	0/38	NIRF imaging is capable of detecting failed grafts and allows for revision of the graft during off-pump CABG surgery. Coronary anatomy in obese patients is more easily visualized.
Takahashi et al,^21^ 2004	Graft patency	Patients	72	Prospective observational study	0/72	NIRF imaging is capable of detecting failed grafts and allows for revision of the graft during off-pump CABG surgery.
Balacumaraswami et al,^22^ 2005	Graft patency	Patients	100	Prospective observational study	80/20	Both NIRF imaging and TTFM are useful for confirming graft patency. TTFM alone causes unnecessary graft revision in 10% of patients.
Desai et al,^23^ 2005	Graft patency	Patients	120	Prospective observational study	116/4	NIRF imaging is capable of detecting graft failure and allows for graft revision during off-pump CABG surgery.
Desai et al,^24^ 2006	Graft patency	Patients	106	RCT	104/2	NIRF imaging provides better diagnostic accuracy than TTFM for detecting clinically significant graft failure.
Handa et al,^27^ 2009	Graft patency	Patients	39	Prospective observational study	0/39	Displaying the fluorescent signal against a natural-colored background improves visibility of abnormal flow and perfusion.
Waseda et al,^28^ 2009	Graft patency	Patients	137	Prospective observational study	0/137	NIRF imaging is capable of detecting failed grafts and allows for revision of the graft during off-pump CABG surgery.
Handa et al,^29^ 2010	Graft patency	Patients	51	Prospective observational study	0/51	Displaying the fluorescent signal against a natural-colored background improves visibility of abnormal flow and perfusion. Combining NIRF imaging and TTFM during surgery results in better graft evaluation than TTFM only.
Singh et al,^30^ 2010	Graft patency	Patients	78	RCT	71/7	Graft patency and clinical outcomes were not different at 1 y for patients who received NIRF imaging and TTFM compared to control patients. Assessment with NIRF imaging and TTFM should be used when clinical suspicion of graft failure exists.
Hassan et al,^32^ 2012	Coronary anatomy, anatomy of harvested graft, graft patency	Dogs	8	Preclinical animal study	0/8	NIRF imaging is a feasible technique for visualizing blood flow in coronary arteries and bypass grafts during TECAB surgery in animals.
Kuroyanagi et al,^33^ 2012	Graft patency	Patients	159	Retrospective observational study	0/159	Delayed inflow of fluorescence may indicate competitive flow from the native coronary artery or even the need for graft revision.
Yamamoto et al,^35^ 2015	Graft patency	Patients	40	Retrospective observational study	4/36	NIRF imaging yields adequate results in predicting graft patency compared to angiography after 1 y. Quantified standards in NIRF imaging are required for optimal results.
Nakamura et al,^39^ 2019	Coronary anatomy, anatomy of harvested graft	Patients	30	Retrospective observational study	0/30	NIRF imaging is feasible for assessing graft flow in robotic MIDCAB surgery.

*NIRF*, Near-infrared fluorescence; *CABG*, coronary artery bypass grafting; *TTFM*, transit time flowmetry; *TECAB*, totally endoscopic coronary artery bypass; *MIDCAB*, minimally invasive direct coronary artery bypass.

### Quantified Assessment in Animals

**Figure 4:**
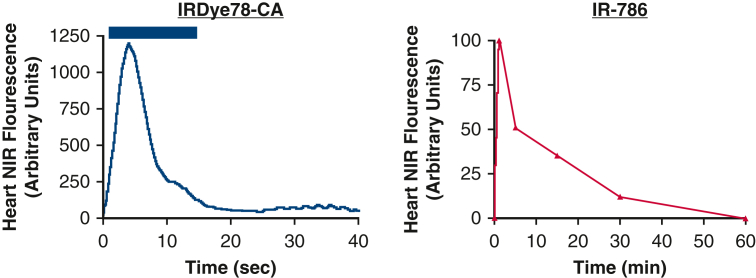
First mention of near-infrared fluorescence quantification in myocardial perfusion assessment.^16^

An objective assessment via quantification of the fluorescent signal has been explored predominantly in animal studies. However, quantification methods and study aims differed notably among the studies. Nakayama and colleagues^16^ were the first to report quantified myocardial perfusion with NIRF imaging in 2002. Their study focused on real-time visualization of myocardial blood flow and administration of cardiac gene therapy with the fluorophores IR-786 and IRDye78-CA in rats. The results demonstrated the possibility of creating a time-intensity curve (Figure 4). However, the author did not use these curves in their subsequent analysis of ischemic areas in the myocardium after infarction. Instead, only visual data for ischemic areas were reported. In 2021, Mashalchi and colleagues^40^ tested a new LED-light guided fluorescence system to assess myocardial perfusion in ex vivo rats, and demonstrated the potential to use quantification to distinguish areas of ischemia following induced occlusion.

Detter and colleagues^25^ used NIRF quantification to assess the degree of coronary artery stenosis in pigs. After inducing various degrees of stenosis in the left anterior descending artery, they created time-intensity curves of the fluorescent signal. Parameters such as peak fluorescence intensity and maximum fluorescence inflow demonstrated the possibility of differentiating between the various stenotic grades (flow reductions of 25%, 50%, and 75%). Wipper and colleagues^36^ further explored the possibility to differentiate between flow-limiting stenosis and non–flow-limiting stenosis. After comparing anatomic stenosis to the actual perfusion of the dependent myocardial tissue, they concluded that the presence of stenosis does not always result in deterioration of perfusion.

To evaluate cardioplegia distribution in pigs, Soltesz and colleagues^26^ added ICG to the cardioplegic solution. Quantification demonstrated the possibility of differentiating the degree of cardioplegia across different myocardial areas and revealed an inferior distribution pattern for retrograde administration through the coronary sinus compared to antegrade delivery.

Detter and colleagues^38^ continued their research on myocardial NIRF imaging and compared NIRF quantification to coronary angiography and TTFM for CABG surgery in pigs. During this study, they were able to create time-intensity curves and perfusion parameters. Analysis of the results demonstrated that both quantified NIRF and TTFM yielded similar sensitivity in detecting different grades of stenosis. This adds to the benefit of having visual assessment as well, a feature not possible with TTFM.

### Quantified Assessment in Patients

Five studies quantified the fluorescent signal in patients, collectively including a total of 332 patients. Gorki and colleagues^31^ aimed to evaluate the difference in cardioplegia distribution after antegrade and retrograde administration, previously investigated by Soltesz and colleagues.^26^ A total of 14 patients were included, of whom only 9 were analyzed owing to technical issues. Quantification of regions of interest revealed perfusion defects around the right and left anterior veins in retrograde-administered cardioplegia.

Ferguson and colleagues^34^ stressed the importance of understanding the functional severity of the stenosis in the target vessel instead of solely the anatomic presence of a stenosis. They constructed an NIRF- and fractional-flow-reserve (FFR)-based perfusion model with which they were able to quantify the effect of CABG on myocardial perfusion in functional stenosis (flow-limiting according to FFR) as well as anatomic stenosis (diagnosed via coronary angiography) in 160 patients. Interestingly, they found competitive flow in 42 of the 165 internal mammary artery grafts, with 95% of those showing no improvement on regional myocardial perfusion. This indicates that although a stenosis might be anatomically significant, it can still supply an adequate amount of blood to the myocardium, rendering the stenosis anatomic but not functional, according to the authors.

In 2022, Buch and colleagues^41^ continued the findings of Ferguson and colleagues and used quantified NIRF imaging as a reference to evaluate the prognostic capabilities of FFR and instantaneous wave-free ratio (iFR). They concluded that functional stenosis severity as measured with FFR and iFR has a complex nonlinear association with an improvement in regional perfusion after CABG. This association was not present between regional perfusion and anatomical stenosis severity as measured with coronary angiography. The change in regional perfusion was measured as a relative change of fluorescent signal intensity after insertion of the graft.

**Figure 5:**
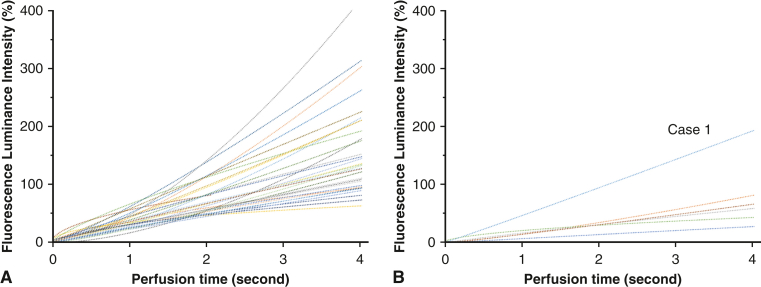
Time-intensity curves of the first 4 seconds after administration of indocyanine green. A, Patent grafts. B, Failed grafts.

Reproduced with permission from Springer Nature.^42^

In their first article, Yamamoto and colleagues^37^ evaluated a quantification technique by retrospectively assessing NIRF recordings to determine graft flow via fluorescence intensity. Subsequently, they compared the results to coronary angiography and TTFM. They identified “average acceleration” (ie, the mean signal velocity) of fluorescence intensity as a useful predictor of graft failure (*P* = .0004). In their second article, Yamamoto and colleagues^42^ again analyzed graft flow according to fluorescence intensity. The difference from their previous study was their use of a camera with a higher resolution to create more accurate time-intensity curves and determine cutoff values. They reported more precise time-intensity curves and found acceptable sensitivity and specificity values for predicting graft failure (83.3% and 69.8%-80.6%, respectively). However, these curves merely demonstrate the first 4 seconds after administration, resulting in the loss of several important parameters of the time-intensity curves, such as the elapsed time to maximum intensity and maximum inflow rate (Figure 5). Cutoff values could not be determined because of the small sample size. Both studies by Yamamoto and colleagues analyzed only the fluorescence intensity in the graft itself, without evaluating the regional blood flow in the perfused myocardial tissue.

An overview of all articles describing quantification in NIRF imaging for myocardial perfusion assessment is provided in Table 2.

**Table 2:** Overview of articles describing quantification in NIRF imaging for myocardial perfusion assessment

Author, y	Goal	Subject	Number	Study design	On/off- pump	Relevant findings
Nakayama et al,^16^ 2002	Myocardial perfusion assessment	Rats	-	Preclinical animal study	-	Possibility to create time-intensity curves from fluorescent signal
Mashalchi et al,^40^ 2021	Coronary anatomy	Rats	-	Preclinical animal study	-	A newly devised LED-light guided fluorescence system is able to quantitatively differentiate ischemic areas of the myocardium.
Detter et al,^25^ 2007	Coronary stenosis	Pigs	11	Preclinical animal study	-	Perfusion parameters can differentiate between different stenotic grades.
Wipper et al,^36^ 2016	Coronary stenosis	Pigs	11	Preclinical animal study	-	Possibility to differentiate between flow-limiting and non–flow-limiting stenosis with perfusion parameters.
Soltesz et al,^26^ 2007	Cardioplegia distribution	Pigs	10	Preclinical animal study	10/0	Quantified NIRF imaging can be used to assess cardioplegia distribution; retrograde cardioplegia delivery yields an inferior distribution pattern compared to antegrade delivery.
Detter et al,^38^ 2018	CABG assessment	Pigs	10	Preclinical animal study	0/10	Quantified NIRF imaging yields similar sensitivity to TTFM in differentiating between different grades of stenosis.
Gorki et al,^31^ 2011	Cardioplegia distribution	Patients	9	Prospective pilot study	9/0	Quantification of the fluorescent cardioplegic signal revealed perfusion defects around the right and left anterior veins in retrograde delivery.
Ferguson et al,^34^ 2013	CABG assessment	Patients	160	Retrospective observational study	55/105	A computed model combining FFR and NIRF imaging can effectively differentiate between anatomic and functional stenoses; competitive flow can be detected following bypass completion.
Buch et al,^41^ 2022	CABG assessment	Patients	41	Prospective pilot study	41/0	Not anatomic, but functional, stenosis severity is associated with change in NIRF signal intensity after CABG.
Yamamoto et al,^37^ 2017	CABG assessment	Patients	69	Retrospective observational study	6/63	“Average acceleration” (mean NIRF signal velocity) is a useful predictor of graft patency.
Yamamoto et al,^42^ 2022	CABG assessment	Patients	43	Retrospective observational study	14/29	More accurate time-intensity curves with improved fluorescence system; acceptable sensitivity and specificity for predicting graft failure

*NIRF*, Near-infrared fluorescence; - , number of subjects not specified; *LED*, light-emitting diode; *CABG*, coronary artery bypass grafting; *TTFM*, transit time flowmetry; *FFR*, fractional flow reserve.

## Discussion

This review of the published research on NIRF imaging for myocardial perfusion assessment shows that the research on qualitative graft assessment demonstrates potential for aiding decision making and improving quality control during CABG surgery. However, the limited amount and heterogenous nature of studies describing quantification indicate that the technological progress of myocardial NIRF imaging has fallen behind. Animal studies have provided valuable information on different techniques and applications for quantification; however, follow-up studies involving patients undergoing CABG are limited. These studies are essential, as they provide valuable information on clinical outcomes attributed to changes in myocardial perfusion after CABG surgery.

The available literature reviews address different techniques used to assess myocardial perfusion.^43^, ^44^, ^45^, ^46^ Regarding NIRF imaging, the need for quantification is commonly discussed as a future research recommendation. Considering this, it is remarkable that only 3 research groups have further evaluated quantification in patients, despite major advancements in other disciplines.^47^^,^^48^

One possible explanation for this lack of research is that specific challenges arise when quantifying the fluorescent signal in open-heart surgery. In on-pump surgery, the fluorescent dye must be administered via cardioplegia, which differs from the standard intravenous route. The possible impact of this alternative administration on the quantified fluorescence signal has not been studied extensively. In off-pump surgery, the motion of the contracting heart continuously affects the intensity of the fluorescent signal, requiring a method to compensate for these motion artifacts. As the techniques to filter for this contraction develop progressively, the opportunities for off-pump NIRF imaging will increase.^49^ This will be an important step, as off-pump surgery most closely resembles the standard physiology of the heart and the peripheral resistance of the capillaries.

Regarding clinical outcomes, Ferguson and colleagues^34^ described a highly relevant capability of quantification. They demonstrated the potential to detect competitive flow with quantified NIRF imaging in More than 25% of patients following completion of the graft anastomosis. Competitive flow regularly results in graft failure in the late postoperative phase (30 days to 1 year) and thus should be prevented.^50^, ^51^, ^52^, ^53^ Combined qualitative and quantitative NIRF assessment could determine the severity of competition between the native coronary artery and the graft. This information could guide the decision making process as, from a theoretical standpoint, ligation of the native coronary artery would eliminate competitive flow and thereby enhance graft performance.

Buch and colleagues^41^ emphasized the importance of basing CABG decisions on functional properties as flow and perfusion rather than on the presence of anatomic stenosis identified through coronary angiography. Intraoperative quantified NIRF imaging, possibly in combination with preoperative FFR and iFR, may provide a comprehensive assessment of the functional performance of coronary arteries and thereby aid in determining which coronary arteries should be targeted for bypass surgery, resulting in a higher clinical success rate per graft.

Several other techniques have been evaluated for their feasibility in myocardial perfusion assessment.^45^^,^^54^^,^^55^ Some of the most promising recent techniques in perfusion imaging include hyperspectral imaging, photoacoustic imaging and laser speckle imaging.^56^, ^57^, ^58^ Initial studies have shown promising results, and these noninvasive techniques have a low threshold for use during interventions. However, at this early stage it is not yet possible to compare the surgical applicability to NIRF imaging and TTFM.

In future studies, systematic assessment of myocardial perfusion with quantified NIRF imaging during CABG surgery should be prioritized. This can be achieved by performing measurements at 2 key points: after opening of the pericardium, to establish baseline myocardial perfusion status, and after completing the distal anastomosis, to assess the immediate changes in myocardial perfusion resulting from bypass. By using a standardized measurement protocol, variable factors such as camera distance and angle, ICG dosage, and measurement duration are eliminated. Subsequently, separate regions of interest can be analyzed for the natural coronary anatomy, graft flow, and perfusion of the myocardial tissue itself. Time-intensity curves and additional inflow and outflow parameters derived from these regions can be correlated to clinical outcomes and systematically evaluated against the predictive values of other techniques, such as TTFM (if conducted). Eventually, this might result in the possibility to establish cutoff values to differentiate between adequate and impaired myocardial perfusion.

## Conclusions

This literature review demonstrates that NIRF imaging for CABG assessment has been broadly researched from a qualitative perspective. The promising results for visualizing perfusion can improve quality control and aid the decision making process during surgery; however, the logical step of further exploration of quantification has been limited, hindering progress in our understanding of the ischemic myocardium. To provide an objective tool best capable of intraoperatively evaluating myocardial perfusion, it is crucial that future studies focus on quantification of NIRF imaging in cardiovascular patients.

## Conflict of Interest Statement

The authors reported no conflicts of interest.

The *Journal* policy requires editors and reviewers to disclose conflicts of interest and to decline handling or reviewing manuscripts for which they may have a conflict of interest. The editors and reviewers of this article have no conflicts of interest.

## Appendix E1 Search Strategy

((“Near-Infrared Imaging”[tw] OR “Near Infrared Imaging”[tw] OR “angiograph∗”[tw] OR “LA-ICG”[tiab] OR “Fluorescen∗”[tw] OR “NIR”[tiab] OR “Infrared Rays”[Mesh] OR “Optical Imaging”[Mesh] OR “Fluorescence”[Mesh] OR “Lasers, Dye”[Mesh] OR “Optical Imaging”[tw] OR “NIR imaging”[tw] OR “Near-infrared fluorescence imaging”[tw] OR “near-infrared”[tw] OR “near-infrared imaging”[tw] OR “NIRF imaging”[tw] OR “Fluorescent Dyes”[Mesh]) AND (“Indocyanine Green”[Mesh] OR “Indocyanine Green”[tw] OR “Wofaverdin”[tw] OR “Vophaverdin”[tw] OR “Vofaverdin”[tw] OR “Cardio-Green”[tw] OR “Cardio Green”[tw] OR “Cardiogreen”[tw] OR “fluorescent cardiac imaging”[tw])) AND (“Myocardial Perfusion Imaging”[Mesh] OR “Perfusion Imaging”[Mesh] OR “Myocardial blood flow”[tw] OR “Myocardial perfusion imaging”[tw] OR “Perfusion Imaging”[tw] OR (“Perfusion”[Mesh] OR “Perfusions”[tw] OR “Perfusion”[tw] AND “Myocardium”[Mesh] OR “Cardiac Muscle”[tw] OR “Heart Muscle”[tw] OR “Heart Muscles”[tw] OR “Myocardia”[tw] OR “Cardiac Muscles”[tw]) OR “Thoracic Surgery”[Mesh] OR “Thoracic Surgery”[tw] OR “Heart”[tw] OR “Cardiac∗”[tw] OR “Cardio∗”[tw] OR “cardiothoracic”[tw] OR “myocard∗”[tw] OR “Cardiac∗”[tiab] OR “graft patency”[tw]).
